# Codon usage of host-specific P genotypes (VP4) in group A rotavirus

**DOI:** 10.1186/s12864-022-08730-2

**Published:** 2022-07-16

**Authors:** Han Wu, Bingzhe Li, Ziping Miao, Linjie Hu, Lu Zhou, Yihan Lu

**Affiliations:** 1 Department of Epidemiology, Ministry of Education Key Laboratory of Public Health Safety (Fudan University), School of Public Health, Fudan University, Shanghai, 200032 China; 2 Institute of Communicable Diseases Prevention and Control, Zhejiang Provincial Center for Disease Control and Prevention, Hangzhou, 310052 Zhejiang China

**Keywords:** Group A rotavirus, P genotype, VP4, Complete coding sequence, Codon usage bias, Evolution, Animal host, Zoonosis

## Abstract

**Background:**

Group A rotavirus (RVA) is a common causative agent of acute gastroenteritis in infants and young children worldwide. RVA P genotypes, determined by VP4 sequences, have been confirmed to infect humans and animals. However, their codon usage patterns that are essential to obtain insights into the viral evolution, host adaptability, and genetic characterization remained unclear, especially across animal hosts.

**Results:**

We performed a comprehensive codon usage analysis of eight host-specific RVA P genotypes, including human RVA (P[4] and P[8]), porcine RVA (P[13] and P[23]), and zoonotic RVA (P[1], P[6], P[7] and P[19]), based on 233 VP4 complete coding sequences. Nucleotide composition, relative synonymous codon usage (RSCU), and effective number of codons (ENC) were calculated. Principal component analysis (PCA) based on RSCU values was used to explore the codon usage patterns of different RVA P genotypes. In addition, mutation pressure and natural selection were identified by using ENC-plot, parity rule 2 plot, and neutrality plot analyses. All VP4 sequences preferred using A/U nucleotides (A: 0.354-0.377, U: 0.267-0.314) than G/C nucleotides across genotypes. Similarly, majority of commonly used synonymous codons were likely to end with A/U nucleotides (A: 9/18-12/18, U: 6/18-9/18). In PCA, human, porcine, and zoonotic genotypes clustered separately in terms of RSCU values, indicating the host-specific codon usage patterns; however, porcine and zoonotic genotypes were partly overlapped. Human genotypes, P[4] and P[8], had stronger codon usage bias, as indicated by more over-represented codons and lower ENC, compared to porcine and zoonotic genotypes. Moreover, natural selection was determined to be a predominant driver in shaping the codon usage bias across the eight P genotypes. In addition, mutation pressure contributed to the codon usage bias of human genotypes.

**Conclusions:**

Our study identified a strong codon usage bias of human RVA P genotypes attributable to both natural selection and mutation pressure, whereas similar codon usage bias between porcine and zoonotic genotypes predominantly attributable to natural selection. It further suggests possible cross-species transmission. Therefore, it warrants further surveillance of RVA P genotypes for early identification of zoonotic infection.

**Supplementary Information:**

The online version contains supplementary material available at 10.1186/s12864-022-08730-2.

## Introduction

Rotavirus is the most common cause of severe diarrheal disease in infants and young children globally. According to World Health Organization, rotavirus is responsible for approximately 453,000 deaths in children under five years of age worldwide yearly [1]. Rotavirus is a spherical, non-enveloped and double-stranded RNA virus belonging to the Reoviridae family, Sedoreovirinae subfamily and Rotavirus genus [2]. The rotavirus genome is approximately 18.5 Kb in size and consists of 11 double-stranded RNA segments, encoding six structural proteins (VP1-4, VP6 and VP7) and six non-structural proteins (NSP1-NSP6). Each segment possesses a single open reading frame except segment 11, which contains two genes [3]. Based on the antigenicity of the VP6 protein, rotavirus genus has been classified into 10 species (group A-J) [4]. Among them, group A rotavirus (RVA) is the main cause of acute dehydrating diarrhea in humans and numerous animal species [5]. Furthermore, VP4 and VP7, the two capsid proteins of rotavirus, are involved in a dual classification system defining P and G genotypes, respectively [2]. Currently, more than 40 G and 50 P genotypes have been reported worldwide [6].

Generally, genetic codons are degenerated, with an amino acid being encoded by more than one codon. During protein synthesis, a species or a gene usually tends to use one or more specific synonymous codons, i.e. codon usage bias [7]. Many studies have considered that different species of viruses selected specific codon usage, possibly as a means of exercising control over the translation of viral proteins [8–13]. Deciphering the extent and causes of viral codon usage bias is essential for viral evolution [14]. Codon usage patterns and corresponding driving forces in some RNA viruses have been reported. For example, Chinese porcine circovirus (PCV), rabies virus (RABV), chikungunya virus (CHIKV), and severe acute respiratory syndrome coronavirus 2 (SARS-CoV-2) exhibited a low codon usage bias; however, the main driving factors were natural selection for PCV and RABV and mutation pressure for CHIKV and SARS-CoV-2 [15–18]. In addition, dinucleotide abundance, tRNA abundance, gene function and length can affect codon usage patterns [16]. Thus, a comprehensive codon usage analysis is of significance to understand the viral evolution, host adaptability, and genetic characterization.

The codon usage related to RVA has also been reported in several studies. One study focused on human G2P[4] found a high codon usage bias of VP4 and VP7 sequences [19]. Another study confirmed that dominance of mutational pressure rather than natural selection accounted for the codon usage bias of avian VP6 sequences [20]. Besides, a study including 789 complete mammalian RVA genomes showed that natural selection and mutation pressure played 81.3% and 18.7% roles in shaping the codon usage bias of VP4, while VP7 was under more selective pressure [21]. Notably, RVA VP4 has distinct hosts according to a previous study [22], compared to other segments. P[4] and P[8] genotypes infect only humans, while P[6] genotype infects both humans and swine. The above three P genotypes are dominant in human rotavirus infections. In addition, swine is a significant animal reservoir of rotavirus P genotypes. Multiple genotypes, such as P[13], P[23], P[26], P[27], P[32] and P[34], infect only swine. In contrast, P[1], P[3], P[7], P[9], P[14], and P[19] infect humans and other animals including swine [22]. However, codon usage patterns of RVA P genotypes remains unclear, especially across animal hosts. 

Therefore, this study aimed to comprehensively determine and compare the host-specific codon usage bias among human, animal, and zoonotic (infects both humans and animals) RVA. Considering that RVA P genotypes should cover identical animals between animal and zoonotic RVA, and the number of complete VP4 coding sequences available in the GanBank, we finally selected eight P genotypes for codon usage analysis. These P genotypes were divided into three groups: human group (P[4] and P[8], infect only humans), porcine group (P[13] and P[23], infect only swine) and zoonotic group (P[1], P[6], P[7] and P[19], infect both humans and swine) [22]. Our findings would facilitate new insights into molecular evolution, host selection and regulation of viral gene expression of RVA.

## Results

### Nucleotide compositions and properties

Nucleotides A and U of VP4 coding sequences of RVA were more abundant than those of G and C (A: 0.354-0.377, U: 0.267-0.314, *P* < 0.01), regardless of P genotypes (Table 1). Similarly, the two more abundant nucleotides at the third position of synonymous codons (A3, U3, G3, C3) were A3 (0.515-0.575) and U3 (0.362-0.497) across the eight P genotypes (*P* < 0.01). In addition, GC1 contents were the highest (0.396-0.441), followed by GC2 and GC3 in all genotypes (*P <* 0.01). Accordingly, human RVA (P[4], P[8]), porcine RVA (P[13], P[23]), and zoonotic RVA (P[1], P[6], P[7], P[19]) shared similar nucleotide compositions and properties of codons. Detailed nucleotide compositions and properties of each P genotype were showed in Additional file 1.

**Table 1:** Nucleotide compositions and properties of VP4 coding sequences for RVA P genotypes

Categories	Human genotypes		Porcine genotypes		Zoonotic genotypes			
	P[4]	P[8]	P[13]	P[23]	P[1]	P[6]	P[7]	P[19]
A	0.374(0.002)	0.377(0.005)	0.361(0.001)	0.359(0.003)	0.366(0.006)	0.354(0.003)	0.360(0.004)	0.357(0.002)
U	0.314(0.001)	0.309(0.002)	0.279(0.002)	0.284(0.001)	0.267(0.001)	0.299(0.003)	0.278(0.005)	0.305(0.002)
G	0.164(0.001)	0.160(0.004)	0.182(0.001)	0.179(0.002)	0.190(0.000)	0.178(0.003)	0.175(0.002)	0.172(0.003)
C	0.148(0.001)	0.154(0.002)	0.178(0.001)	0.179(0.001)	0.177(0.005)	0.170(0.002)	0.187(0.003)	0.167(0.002)
AU	0.688(0.002)	0.686(0.005)	0.641(0.002)	0.643(0.003)	0.633(0.005)	0.653(0.004)	0.638(0.002)	0.662(0.004)
GC	0.312(0.002)	0.314(0.005)	0.359(0.002)	0.357(0.003)	0.367(0.005)	0.348(0.004)	0.362(0.002)	0.338(0.004)
GC1	0.396(0.003)	0.395(0.004)	0.435(0.005)	0.433(0.004)	0.441(0.003)	0.425(0.005)	0.431(0.004)	0.410(0.001)
GC2	0.384(0.002)	0.385(0.003)	0.414(0.002)	0.419(0.002)	0.410(0.003)	0.390(0.006)	0.424(0.002)	0.394(0.003)
GC3	0.157(0.005)	0.162(0.014)	0.229(0.003)	0.220(0.005)	0.251(0.009)	0.228(0.007)	0.231(0.005)	0.211(0.011)
A3	0.558(0.004)	0.575(0.016)	0.567(0.002)	0.571(0.008)	0.574(0.021)	0.525(0.009)	0.558(0.018)	0.515(0.007)
U3	0.497(0.006)	0.475(0.006)	0.387(0.005)	0.393(0.004)	0.362(0.008)	0.436(0.008)	0.393(0.013)	0.461(0.008)
G3	0.137(0.004)	0.126(0.018)	0.178(0.005)	0.182(0.008)	0.210(0.005)	0.177(0.008)	0.166(0.010)	0.162(0.010)
C3	0.076(0.007)	0.091(0.006)	0.124(0.003)	0.108(0.003)	0.121(0.007)	0.124(0.007)	0.134(0.006)	0.117(0.008)

All values were displayed in mean (std)

### Host-specific codon usage patterns

**Fig. 1:**
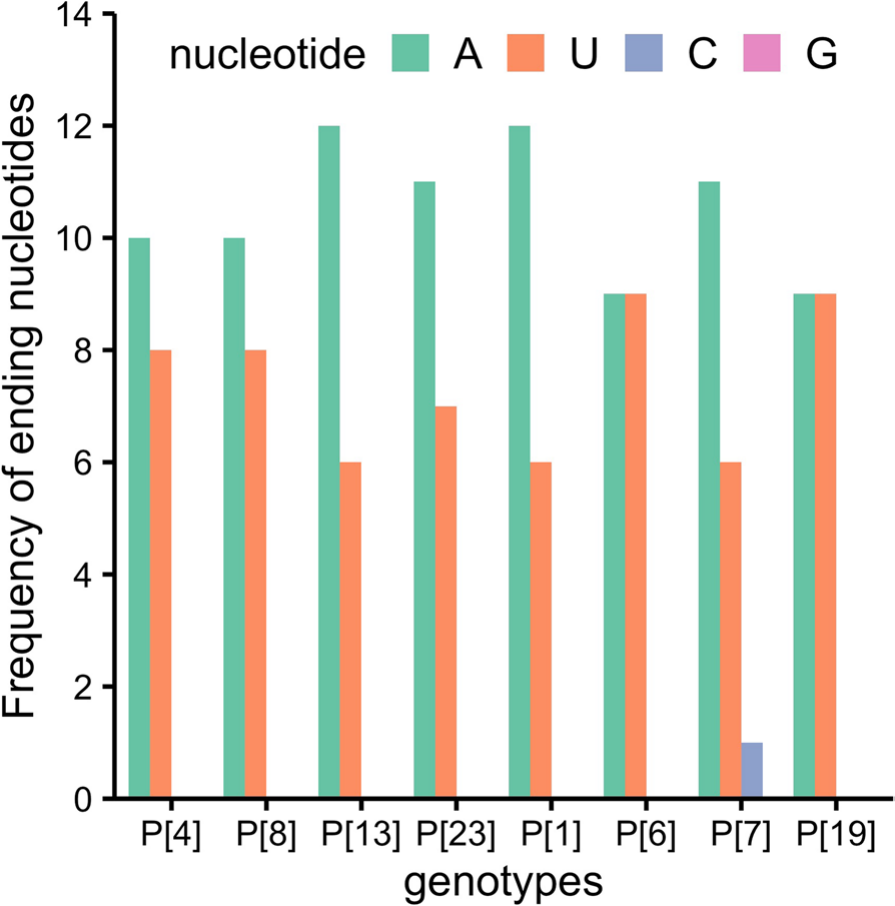
Frequency of ending nucleotides across the 18 preferred synonymous codons for eight RVA P genotypes

Relative synonymous codon usage (RSCU) values were calculated to determine the codon usage pattern of each P genotype. Among the 18 most commonly used synonymous codons, all the eight genotypes, except P[7], ended with A or U nucleotide (Fig. 1). Moreover, 15 and 16 of the 18 codons were over-represented (RSCU>1.6) in P[4] and P[8] genotypes (human genotypes), respectively, which were remarkably high. In regards to the other genotypes, P[13] and P[23] (porcine genotypes), had 8 and 9 most used codons with RSCU values > 1.6, respectively; P[1], P[6], P[7] and P[19] (zoonotic genotypes) had 11, 11, 8 and 12 most used codons with RSCU values > 1.6, respectively (Table 2). Majority of the most used codons of human genotypes had RSCU values > 1.6, suggesting a strong codon usage bias. Detailed RSCU values of each P genotype were displayed in Additional file 2.

**Table 2:** Number of most commonly used codons with RSCU values > 1.6 for RVA P genotypes

Group	Genotypes	RSCU value > 1.6
Human	P[4]	15
	P[8]	16
Porcine	P[13]	8
	P[23]	9
Zoonotic	P[1]	11
	P[6]	11
	P[7]	8
	P[19]	12

*RSCU* relative synonymous codon usage

**Fig. 2:**
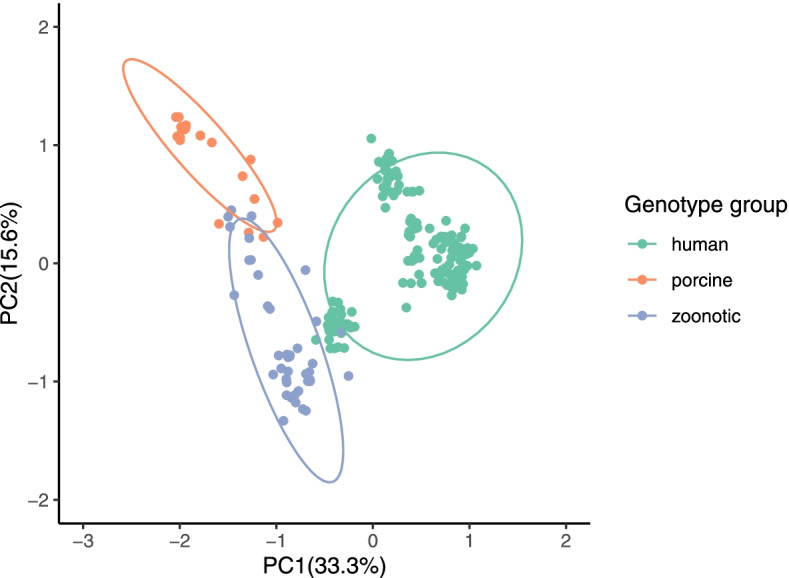
Principal component analysis (PCA) of VP4 coding sequences for eight RVA P genotypes. The eight P genotypes were classified into three groups by hosts: human, porcine and zoonotic groups. X and Y axis represented principal component 1 and principal component 2, respectively. The dots represented VP4 sequences. The ellipses in the figure predicted new observations with a probability of 0.95. New observations from the same group were expected to fall inside the ellipses

In the principal component analysis (PCA) analysis, principal component 1 and principal component 2 explained 33.3% and 15.6% of the total RSCU variance, respectively (Additional file 3). The sequences of human, porcine and zoonotic genotypes formed three distinct clusters (Fig. 2), implying the host-specific codon usage patterns across the RVA P genotypes. However, the predicting ellipses of the sequences of porcine and zoonotic genotypes were overlapped. This indicated some similarities in the codon usage pattern, which may be a clue to the cross-species transmission.

### Codon usage bias

By calculating the effective number of codons (ENC) values of RVA VP4 sequences, we estimated the codon usage bias. ENC values of human genotypes (38.18±0.54 and 37.86±1.08 for P[4] and P[8], respectively) were lower than those of porcine (42.93±0.73 and 42.23±0.80 for P[13] and P[23], respectively) and zoonotic genotypes (41.26±0.40, 42.24±1.01, 43.02±0.37 and 41.75±0.91 for P[1], P[6], P[7] and P[19], respectively), indicating a stronger codon usage bias in the human genotypes (*P* < 0.05), which was consistent with the RSCU analysis (Table 3).

**Table 3:** ENC values of VP4 complete coding sequences for RVA P genotypes

Group	Genotypes	Mean ± std	Range
Human	P[4]	38.18 ± 0.54	36.79-39.17
	P[8]	37.86 ± 1.08	35.99-39.97
Porcine	P[13]	42.93 ± 0.73	42.14-43.57
	P[23]	42.23 ± 0.80	40.38-43.26
Zoonotic	P[1]	41.26 ± 0.40	40.98-41.54
	P[6]	42.24 ± 1.01	40.24-43.99
	P[7]	43.02 ± 0.37	42.40-43.48
	P[19]	41.75 ± 0.91	40.71-42.33

*ENC* effective number of codons

### Forces driving codon usage bias

**Fig. 3:**
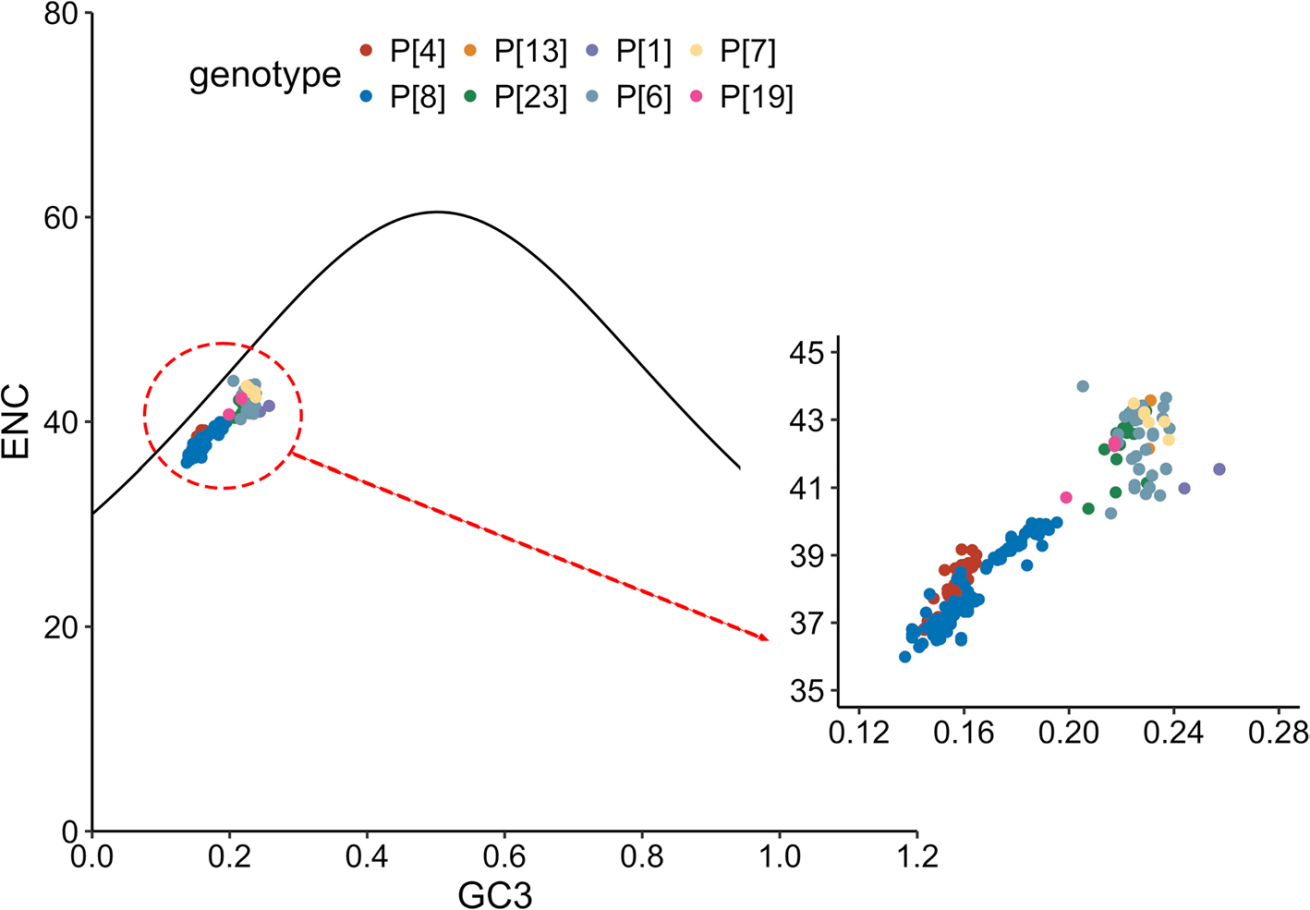
ENC-plot analysis of VP4 coding sequences for the eight RVA P genotypes. The ENC values were plotted against the GC contents at the third codon position (GC3). The expected curve represented the expected ENC values according to corresponding GC3 contents. The dots represented VP4 sequences

**Fig. 4:**
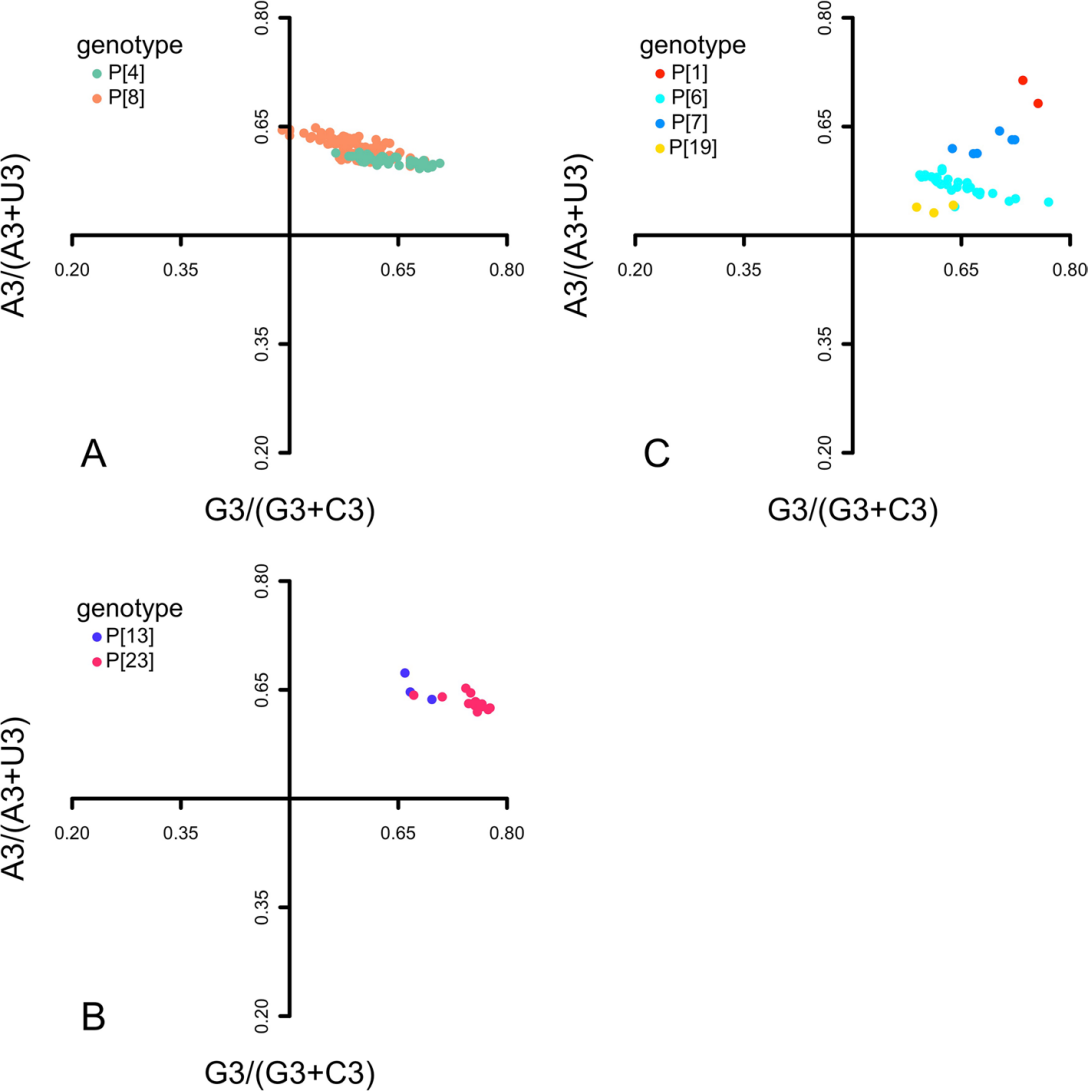
Parity rule 2 (PR2) analysis of VP4 coding sequences for eight RVA P genotypes. **A** Human genotypes. **B** Porcine genotypes. **C** Zoonotic genotypes. A3/(A3 + U3) and G3/(G3 + C3) of fourfold degenerate codon families represented the ordinate and abscissa, respectively. In the centre of the graph, both coordinates had a value of 0.5, i.e. A = U and G = C. The dots represented VP4 sequences

In ENC-plot analysis, ENC values were plotted against GC3 values to explore the forces driving codon usage bias (Fig. 3). We found that all the VP4 sequences fell below the expected ENC curve and clustered together. This revealed that natural selection also played roles in codon usage bias, in addition to mutation pressure, regardless of P genotypes. To further estimate the effects of mutation pressure and natural selection, parity rule 2 (PR2) analysis was performed (Fig. 4). All the sequences were away from the origin (0.5, 0.5). Accordingly, mutation pressure and natural selection both contributed to the codon usage bias for all genotypes; however, these two factors had different impacts.

**Fig. 5:**
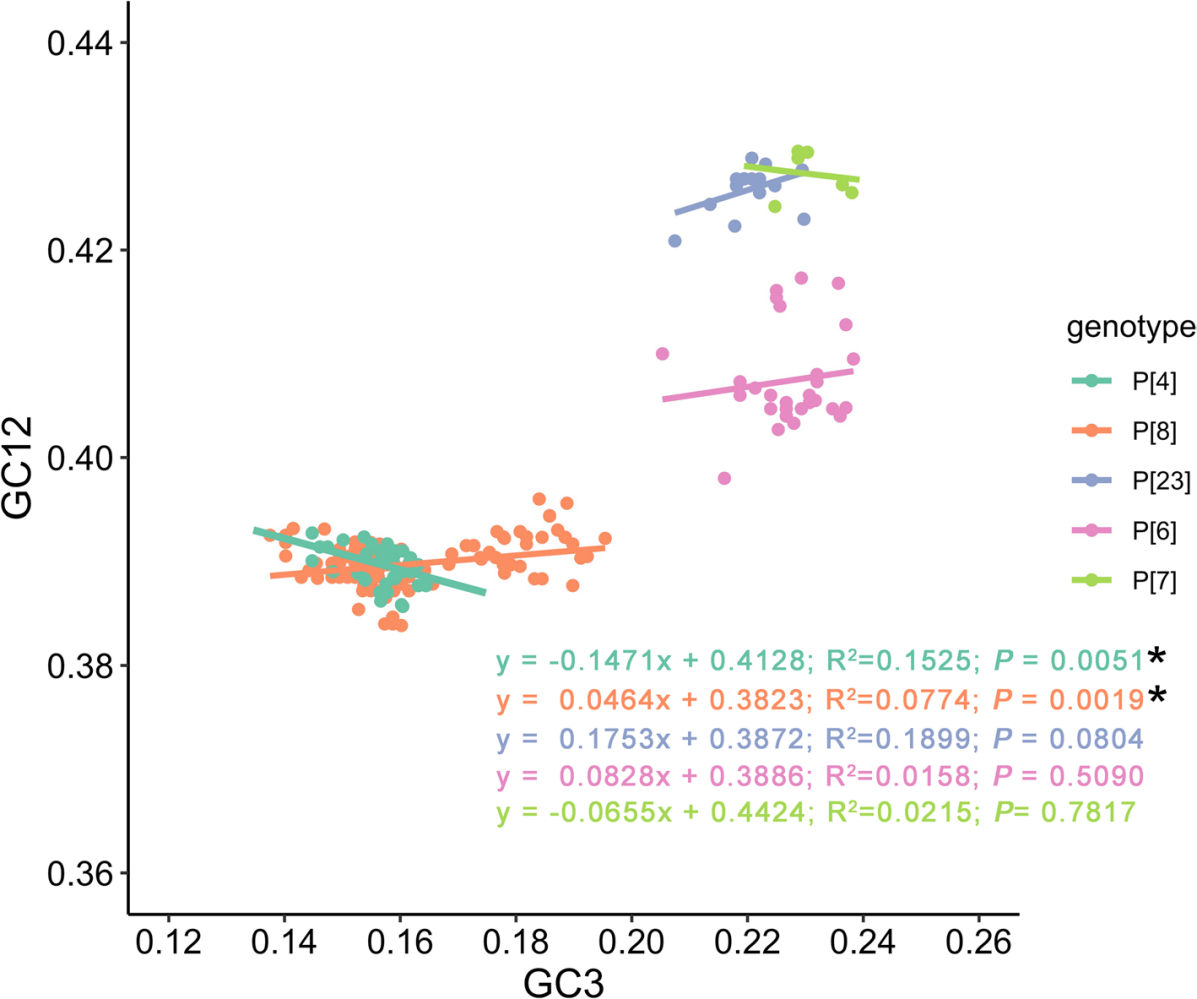
Neutrality plot analysis of VP4 coding sequences for eight RVA P genotypes. The GC12 values were plotted against the GC3 values. The dots represented VP4 sequences. * Represented correlation significant at *P* < 0.05

Moreover, neutrality plot analysis was performed to compare the contributions to codon usage bias between natural selection and mutation pressure. P[1], P[13] and P[19] genotypes were excluded from the neutrality plot analysis due to the limited number of VP4 sequences (*n *= 2, *n *= 3 and *n *= 3, respectively). The correlation between GC12 and GC3 was calculated (Fig. 5). In human group, the slopes of the regression line were -0.1471 (*P* = 0.0051) and 0.0464 (*P* = 0.0019) for P[4] and P[8] genotypes, respectively, suggesting the constraint of 14.71% and 4.64% by mutation pressure, and 85.29% and 95.36% by natural selection, respectively, in shaping the codon usage bias. In contrast, for porcine and zoonotic groups, there was no significant correlation between GC12 and GC3 (P[23]: *P =* 0.0804, P[6]: *P* = 0.5090, P[7]: *P* = 0.7817), indicating natural selection totally driving the codon usage bias [23]. Accordingly, natural selection played a major role in shaping the codon usage bias, regardless of P genotypes.

## Discussion

Currently, phylogenetic analysis is well studied to explore the evolution of RVA [24–26]. Here, we carried out a systematic and comprehensive codon usage analysis of RVA VP4 coding sequences across the eight P genotypes, covering human, porcine and zoonotic genotypes, to demonstrate the viral evolution from a new perspective. So far, more than 50 P genotypes have been identified [6]; however, host species have been documented in only 35 P genotypes [22]. In our study, we aimed to determine the host-specific codon usage bias across humans and animals, so we included P genotypes that isolated only in humans, only in certain animals, and in both humans and identical animals. Considering the VP4 sequences available for analysis, we finally included eight RVA P genotypes in the study, including P[4] and P[8] (human genotypes), P[13] and P[23] (porcine genotypes), and P[1], P[6], P[7, 19] genotypes (zoonotic genotypes that were isolated in both humans and swine). We found that all RVA VP4 coding sequences, regardless of P genotypes, preferred using A and U nucleotides. Similarly, most commonly used synonymous codons were likely to end with A/U in RSCU analysis. Furthermore, three distinct clusters were found in PCA across the eight P genotypes, indicating different evolutionary groups. However, there were some overlaps in the predicted area of the sequences of porcine and zoonotic genotypes, suggesting possible cross-species transmission. Based on ENC analysis, human genotypes had higher codon usage bias compared to porcine and zoonotic genotypes. Morever, natural selection was a predominant driver in shaping the codon usage bias across the eight P genotypes through ENC-plot, PR2, and neutrality plot analyses.

Nucleotide composition bias is possibly caused by mutational bias or selection for function [27, 28] or evasion of innate immune system [29, 30]. Similar to RVA VP4 coding sequences, sequences of many other RNA viruses, such as transmissible gastroenteritis virus (TGEV), porcine deltacoronavirus (PDCoV) and SARS-CoV-2 [23, 31, 32], were also AU-rich. However, sequences of rubella virus were GC-rich [8]. A previous study found that the biased nucleotide composition (A-rich) of human immunodeficiency virus-1 may be related to its pathogenicity [30]. Thus, it warrants further study to explore the association with RVA pathogenicity.

Codon usage bias leads to different levels of translation efficiency, with highly expressed genes showing stronger bias for codons [33, 34]. It has been reported that RVA codon usage patterns appeared optimally for expression in humans and birds, compared to other hosts [21]. It may explain why the two human genotypes, P[4] and P[8], had stronger codon usage bias and had the most over-represented preferred codons, compared to porcine and zoonotic genotypes. However, porcine genotypes (ENC = 42.34±0.81) and zoonotic genotypes (ENC = 42.27±0.98) remained moderate codon usage bias, compared to other viruses including Ebola virus (ENC = 57.23±0.51), SARS-CoV-2 (ENC = 48.54±2.34), and Middle East respiratory syndrome coronavirus (ENC = 49.82±0.08) [11, 35, 36]. Low codon usage bias, which contribute to more efficient viral replication and overcoming host defense mechanisms, allows persistent infection in optimized host [8, 16]. These findings may illustrate why RVA have a diversity of P genoptyes and a broad range of hosts, which has raised a concern of zoonotic transmission.

Mutation pressure and natural selection, the two main factors accounting for codon usage bias, exist in genes of different organisms [37]. Generally, for RNA viruses, mutation pressure was found to be the predominant factor compared with natural selection, as RNA viruses have a higher mutation rate [37, 38]. However, our findings revealed that natural selection was determined to be a principal driver in shaping the codon usage bias across the eight RVA P genotypes, which was consistent with some other viruses, such as TGEV, PDCoV and Zika virus [23, 31, 39]. We suggested that the dominant selection pressure was responsible for the rapid rate of viral evolution, resulting in a diversity of genotypes. The mechanisms of the imbalance between natural selection and mutation pressure need further study.

Our study had some strengths. Firstly, we selected a variety of host-specific RVA P genotypes, covering human, porcine and zoonotic genotypes. The comparison among the P genotypes with different hosts may explore the possible cross-species transmission with a perspective of codon usage. Secondly, the sequences included in the study were complete VP4 coding sequences, containing more biological information. Thirdly, we used multiple methods, including nucleotide composition, RSCU, ENC-plot, PR2 analyses, and so on, to comprehensively demonstrate codon usage bias and driving forces. However, the study had also limitations. Some genotypes, such as P[1], P[13] and P[19], have few full-length sequences, which might result in unreliable findings in the codon usage analysis. In addition, we included only porcine genotypes and zoonotic genotypes that infects swine in the study among multiple RVA animal genotypes, due to the data availability in Genbank. Thus, the further epidemiological surveillance is essential to learn more about RVA.

## Conclusions

The RVA VP4 coding sequences were AU-rich, regardless of P genotypes. However, human genotypes, P[4] and P[8], had stronger codon usage bias that was shaped by both natural selection and mutation pressure. In contrast, porcine and zoonotic genotypes (P[13], P[23], P[1], P[6], P[7] and P[19]) shared similar codon usage bias, in which natural selection was a predominant driver. Furthermore, it may be attributable to possible cross-species transmission. Therefore, it warrants further surveillance of RVA P genotypes, which may facilitate early identification of zoonotic infection.

## Methods

### Sequence collection

Currently, a total of 35 RVA P genotypes had specific hosts [22]. This study aimed to determine the host-specific codon usage bias across humans and animals, so we included P genotypes that isolated only in humans, only in certain animals, and in both humans and identical animals. We included P genotypes due to following criteria: 1) qualified sequences, which was defined as a VP4 full-length sequence, without undetermined codon (X, W, K, and R), and not a vaccine-related sequences; and 2) number of qualified sequences for each host within each P genotype was ≥ 2. Finally, we included eight RVA P genotypes in the study. Complete VP4 coding sequence of RVA P[4], P[8], P[13], P[23], P[1], P[6], P[7] and P[19] genotypes were retrieved from the GenBank of the National Center for Biotechnological Information available through 21 April 2022 [40]. The total number of P[4], P[8], P[13], P[23], P[1], P[6], P[7] and P[19] sequences were 128, 593, 5, 17, 2, 31, 10, and 3, respectively. In order to remove redundancy of P[4] and P[8] sequences, some sequences were randomly excluded due to same collection year with an identity ≥ 98% at the nucleotide level. Consequently, a total of 233 VP4 coding sequences were included in the analysis, of which three groups, human RVA, porcine RVA, and zoonotic RVA, were classified (Table 4). The detailed sequence information (accession number, strain name, P genotype, host, country, and collection year) were displayed in Additional file 4.

**Table 4:** Basic information of VP4 coding sequences for RVA P genotypes

Group	Genotypes	Host	Number of sequences
Human	P[4]	Human	50
	P[8]		122
Porcine	P[13]	Swine	3
	P[23]		17
Zoonotic	P[1]	Human, swine and other animals	2
	P[6]	Human and swine	30
	P[7]	Human, swine and bovine	6
	P[19]	Human and swine	3

### Nucleotide composition analysis

Nucleotide composition (A%, U%, C%, G% , AU% and GC%) of whole codons and at the third position (A3%, U3%, C3%, G3%) were calculated by MEGA 11 [41] and Codon W 1.4.2 (http://codonw.sourceforge.net/), respectively. The GC contents in synonymous codons at each position (GC1%, GC2% and GC3%) were calculated using Emboss: cusp [42]. The five codons (AUG, UAG: only encode for Met and Trp amino acids, respectively; UAA, UAG, UGA: termination codons) that do not lead to usage bias were removed from the codon usage analysis. One-way analysis of variance (ANOVA) was utilized for the comparsion of nucleotide compositions and other nucleotide properties. A *P* value < 0.05 was considered statistically significant.

### Relative synonymous codon usage (RSCU)

RSCU is the observed frequencies divided by that expected if usage of synonymous codons is unbiased. The RSCU is calculated as:

1 $${\mathrm{RSCU}}_{\mathrm{i}\mathrm{j}}=\frac{{\mathrm{X}}_{\mathrm{i}\mathrm{j}}}{\sum_{\mathrm{j}=1}^{{\mathrm{n}}_{\mathrm{i}}}\ {\mathrm{X}}_{\mathrm{i}\mathrm{j}}}\ {\mathrm{n}}_{\mathrm{i}}$$

where xij is the number of occurrence of the jth codon for the ith amino acid encoding by ni synonymous codons [43]. RSCU = 1 , > 1 and < 1 indicate absent, positive, and negative codon bias, respectively. In addition, RSCU > 1.6 or < 0.6 indicates high or low expression of the synonymous codon [44]. MEGA 11 was used to calculated RSCU values [41].

### Principal component analysis (PCA)

PCA is a widely used data dimension reduction method to simplify the simultaneous interpretation of a number of related variables [45]. In this study, the RSCU values of the 59 codons of each sequence (with AUG, UGG and three stop codons removed) formed 59-dimensional vector, which was converted into two composite variables (i.e. principal component 1 and principal component 2). This removed redundant information and make the results easier to understand. PCA was performed using “psych” package [46] of R 4.1.1 (https://www.r-project.org/).

### Effective number of codons (ENC)

ENC values reflect the extent of codon preference in a gene, which range from 20 (only one codon used per amino acid) through 61 (all codons used equally), with smaller ENC values indicating stronger codon usage bias [47]. In general, an ENC value of less than or equal to 40 indicates a strong codon usage bias [48]. The ENC values are calculated as:

2 $$\mathrm{ENC}=2+\frac9{{\overline{\mathrm F}}_2}+\frac1{{\overline{\mathrm F}}_3}+\frac5{{\overline{\mathrm F}}_4}+\frac3{{\overline{\mathrm F}}_6}$$

where ${\overline{\mathrm{F}}}_{\mathrm{i}}$ (i = 2, 3, 4, 6) represents the average value of ${\overline{\mathrm{F}}}_{\mathrm{i}}$ for i-fold degenerate codon families. Using the follwing formula to calculate ${\overline{\mathrm{F}}}_{\mathrm{i}}$ value:

3 $${\overline{\mathrm{F}}}_{\mathrm{i}}=\frac{\mathrm{n}{\sum}_{\mathrm{j}=1}^{\mathrm{i}}{\left(\frac{n_j}{n}\right)}^2-1}{\mathrm{n}\hbox{-} 1}$$

where n represents the whole number of occurrence of the codons for that amino acid and n_j_ is the number of occurrence of the jth codon for that amino acid. Furthermore, one-way ANOVA was utilized to test ENC difference among genotypes. A *P* value < 0.05 was considered statistically significant.

### ENC-plot analysis

The ENC values are plotted against the GC3 values of each sequence in ENC-plot analysis. The expected ENC values are calculated using the following formula:

4 $${\mathrm{ENC}}_{\exp \mathrm{ected}}=2+\mathrm{s}+\frac{29}{s^2+{\left(1-\mathrm{s}\right)}^2}$$

where s denotes the GC3. When the codon usage bias is influenced only by mutation pressure, the ENC values will fall on the expected curve. If the actual ENC values fall below the expected curve, then the codon usage bias is drived by other factors such as natural selection in addition to mutation pressure [16].

### Parity rule 2 (PR2) analysis

PR2 is performed to explore the effects of mutation pressure and natural selection on codon usage bias. The AU bias [A3/(A3 + U3)] and GC bias [G3/(G3 + C3)] of fourfold degenerate codon families (alanine, arginine, glycine, leucine, proline, serine, threonine and valine) represent the ordinate and abscissa, respectively. In the centre of the graph, both coordinates have a value of 0.5, i.e. A = U and G = C, indicating that mutation pressure and natural selection are equal [49]. A value of bias greater than 0.5 indicates the preference of purine over pyrimidine, and vice versa, which means deviation between the mutation pressure and natural selection [31, 50].

### Neutrality plot analysis

Neutrality plot analysis is used to demonstrate the effects of mutation pressure and natural selection on codon usage bias [51]. The GC12 contents are plotted against the GC3 contents. The contributions of mutation pressure and natural selection are tested by the regression slope between GC12 and GC3. Mutaton pressure plays a major role on codon usage bias if the regression slope is statistically significant and close to 1. Codon usage bias is completely drived by natural selection if the regression slope = 0 or is not statistically significant [23, 51]. Correlation between GC12 and GC3 was calculated by simple linear regression. A *P* value < 0.05 was considered statistically significant.

## Supplementary Information

**Additional file 1: Table S1.** Nucleotide compositions and properties of VP4 coding sequences for group A rotavirus P[4], P[8], P[13], P[23], P[1], P[6], P[7] and P[19].

**Additional file 2:** **Table S2.** Relative synonymous codon usage (RSCU) patterns of VP4 coding sequences for group A rotavirus P[4], P[8], P[13], P[23], P[1], P[6], P[7] and P[19].

**Additional file 3:** **Figure S1.** Scree plot of percentage of explained variances for each principal component of the relative synonymous codon usage (RSCU) values of group A rotavirus VP4 coding sequences. This plot showed the proportion of variance in the RSCU values for each principal component (dimension), in descending order of magnitude.

**Additional file 4:** **Table S3.** VP4 coding sequences for group A rotavirus P[4], P[8], P[13], P[23], P[1], P[6], P[7] and P[19].

## Data Availability

The total 233 complete RVA VP4 coding sequences used in this study are available in the GenBank of the National Center for Biotechnological Information (https://www.ncbi.nlm.nih.gov/genbank/). The accession numbers of all sequences are showed in Additional file 4.

## Abbreviations

A, U, C, G: adenine, uracil, cytosine, guanine
ENC: effective number of codons
PCA: principal component analysis
PR2: parity rule 2
RSCU: relative synonymous codon usage
RVA: Group A rotavirus
