# Supplementary material for: Codon usage of host-specific P genotypes (VP4) in group A rotavirus
Source: BMC Genomics. 2022 Jul 16;23:518. doi: 10.1186/s12864-022-08730-2 (PMC9288207; doi:10.1186/s12864-022-08730-2)
Supplement: Supplementary file 2 — Additional file 2: Table S2. Relative synonymous codon usage (RSCU) patterns of VP4 coding sequences for group A rotavirus P[4], P[8], P[13], P[23], P[1], P[6], P[7] and P[19]. [file 12864_2022_8730_MOESM2_ESM.docx]

**Table S2. Relative synonymous codon usage (RSCU) patterns of VP4 coding sequences for group A rotavirus P[4], P[8], P[13], P[23], P[1], P[6], P[7] and P[19].**

| **Codon** | **Human genotypes** | |  | **Porcine genotypes** | |  | **Zoonotic genotypes** | | | |
| --- | --- | --- | --- | --- | --- | --- | --- | --- | --- | --- |
|  | **P[4]** | **P[8]** |  | **P[13]** | **P[23]** |  | **P[1]** | **P[6]** | **P[7]** | **P[19]** |
| UUU(F) | **1.58** | **1.69** |  | **1.43** | **1.40** |  | **1.48** | **1.43** | **1.19** | **1.30** |
| UUC(F) | 0.42 | 0.31 |  | 0.57 | 0.60 |  | 0.52 | 0.57 | 0.81 | 0.70 |
| UUA(L) | **3.15** | **3.22** |  | **2.73** | **2.76** |  | **2.09** | **3.05** | **2.71** | **2.91** |
| UUG(L) | 0.90 | 0.85 |  | 0.98 | 0.58 |  | 1.36 | 1.00 | 0.76 | 0.93 |
| CUU(L) | 0.87 | 0.62 |  | 0.71 | 0.58 |  | 0.63 | 0.58 | 0.89 | 0.72 |
| CUC(L) | 0.23 | 0.33 |  | 0.30 | 0.02 |  | 0.21 | 0.32 | 0.15 | 0.31 |
| CUA(L) | 0.70 | 0.87 |  | 0.84 | 1.57 |  | 1.20 | 0.81 | 1.13 | 0.86 |
| CUG(L) | 0.17 | 0.11 |  | 0.44 | 0.49 |  | 0.52 | 0.23 | 0.36 | 0.27 |
| AUU(I) | **1.83** | **1.78** |  | 1.16 | 1.04 |  | 1.09 | 1.32 | 1.30 | 1.25 |
| AUC(I) | 0.13 | 0.12 |  | 0.33 | 0.16 |  | 0.19 | 0.30 | 0.28 | 0.25 |
| AUA(I) | 1.04 | 1.11 |  | **1.52** | **1.80** |  | **1.72** | **1.39** | **1.42** | **1.49** |
| GUU(V) | 1.20 | 1.06 |  | 1.25 | 0.92 |  | 1.04 | **1.52** | 1.27 | **1.77** |
| GUC(V) | 0.26 | 0.27 |  | 0.53 | 0.24 |  | 0.37 | 0.34 | 0.45 | 0.48 |
| GUA(V) | **1.96** | **2.06** |  | **1.54** | **1.99** |  | **1.82** | 1.22 | **1.51** | 1.24 |
| GUG(V) | 0.58 | 0.61 |  | 0.69 | 0.85 |  | 0.77 | 0.93 | 0.78 | 0.51 |
| UCU(S) | 1.07 | 1.06 |  | 1.14 | 1.36 |  | 0.61 | 1.12 | 1.10 | 1.22 |
| UCC(S) | 0.19 | 0.29 |  | 0.22 | 0.22 |  | 0.23 | 0.41 | 0.26 | 0.07 |
| UCA(S) | **2.63** | **3.07** |  | **2.76** | **2.75** |  | **3.63** | **2.69** | **2.92** | **2.78** |
| UCG(S) | 0.73 | 0.43 |  | 0.89 | 0.99 |  | 0.54 | 0.56 | 0.72 | 0.77 |
| AGU(S) | 1.19 | 0.84 |  | 0.67 | 0.42 |  | 0.73 | 0.98 | 0.61 | 1.00 |
| AGC(S) | 0.18 | 0.32 |  | 0.31 | 0.26 |  | 0.27 | 0.24 | 0.38 | 0.16 |
| CCU(P) | 0.82 | 0.76 |  | 0.57 | 0.55 |  | 0.29 | 0.32 | 0.35 | 0.75 |
| CCC(P) | 0.16 | 0.10 |  | 0.18 | 0.03 |  | 0.00 | 0.23 | 0.17 | 0.13 |
| CCA(P) | **2.86** | **2.92** |  | **2.80** | **2.98** |  | **2.91** | **2.88** | **3.14** | **2.46** |
| CCG(P) | 0.16 | 0.23 |  | 0.46 | 0.44 |  | 0.80 | 0.56 | 0.35 | 0.66 |
| ACU(T) | **1.75** | **1.78** |  | 1.03 | 1.34 |  | 1.25 | **1.83** | 1.32 | **1.73** |
| ACC(T) | 0.37 | 0.37 |  | 0.29 | 0.17 |  | 0.17 | 0.25 | 0.30 | 0.49 |
| ACA(T) | 1.48 | 1.49 |  | 1.94 | **1.59** |  | **1.80** | 1.25 | **1.66** | 1.18 |
| ACG(T) | 0.40 | 0.35 |  | 0.74 | 0.90 |  | 0.78 | 0.67 | 0.72 | 0.60 |
| GCU(A) | 1.52 | 1.44 |  | 1.38 | **1.85** |  | 1.12 | **1.67** | **1.71** | 1.47 |
| GCC(A) | 0.22 | 0.19 |  | 0.20 | 0.48 |  | 0.39 | 0.31 | 0.20 | 0.35 |
| GCA(A) | **1.73** | **1.91** |  | **1.77** | 1.29 |  | **1.72** | 1.46 | 1.66 | **1.82** |
| GCG(A) | 0.54 | 0.46 |  | 0.65 | 0.38 |  | 0.77 | 0.56 | 0.43 | 0.35 |
| UAU(Y) | **1.73** | **1.71** |  | **1.59** | **1.50** |  | **1.62** | **1.49** | **1.36** | **1.49** |
| UAC(Y) | 0.27 | 0.29 |  | 0.41 | 0.50 |  | 0.38 | 0.51 | 0.64 | 0.51 |
| CAU(H) | **1.77** | **1.55** |  | **1.33** | **1.15** |  | **1.60** | **1.67** | 0.96 | **1.62** |
| CAC(H) | 0.23 | 0.45 |  | 0.67 | 0.85 |  | 0.40 | 0.33 | **1.04** | 0.38 |
| CAA(Q) | **1.48** | **1.67** |  | **1.51** | **1.34** |  | **1.33** | **1.63** | **1.55** | **1.47** |
| CAG(Q) | 0.52 | 0.33 |  | 0.49 | 0.66 |  | 0.67 | 0.37 | 0.45 | 0.53 |
| AAU(N) | **1.85** | **1.69** |  | **1.50** | **1.66** |  | **1.41** | **1.55** | **1.42** | **1.61** |
| AAC(N) | 0.15 | 0.31 |  | 0.50 | 0.34 |  | 0.59 | 0.45 | 0.58 | 0.39 |
| AAA(K) | **1.70** | **1.58** |  | **1.51** | **1.48** |  | **1.49** | **1.30** | **1.56** | **1.47** |
| AAG(K) | 0.30 | 0.42 |  | 0.49 | 0.52 |  | 0.51 | 0.70 | 0.44 | 0.53 |
| GAU(D) | **1.75** | **1.62** |  | **1.48** | **1.43** |  | **1.26** | **1.49** | **1.54** | **1.63** |
| GAC(D) | 0.25 | 0.38 |  | 0.52 | 0.57 |  | 0.74 | 0.51 | 0.46 | 0.37 |
| GAA(E) | **1.55** | **1.66** |  | **1.59** | **1.58** |  | **1.49** | **1.63** | **1.48** | **1.51** |
| GAG(E) | 0.45 | 0.34 |  | 0.41 | 0.42 |  | 0.51 | 0.37 | 0.52 | 0.49 |
| UGU(C) | **1.72** | **1.97** |  | **1.43** | **1.26** |  | **1.40** | **1.70** | **1.33** | **1.83** |
| UGC(C) | 0.28 | 0.03 |  | 0.57 | 0.74 |  | 0.60 | 0.30 | 0.67 | 0.17 |
| CGU(R) | 0.58 | 0.80 |  | 0.64 | 0.58 |  | 0.46 | 0.65 | 0.40 | 0.48 |
| CGC(R) | 0.21 | 0.06 |  | 0.15 | 0.03 |  | 0.18 | 0.49 | 0.08 | 0.16 |
| CGA(R) | 0.80 | 0.74 |  | 0.79 | 0.77 |  | 0.28 | 0.71 | 0.95 | 0.74 |
| CGG(R) | 0.15 | 0.15 |  | 0.25 | 0.51 |  | 0.28 | 0.08 | 0.43 | 0.21 |
| AGA(R) | **3.70** | **3.35** |  | **3.34** | **3.71** |  | **3.97** | **3.10** | **3.23** | **3.56** |
| AGG(R) | 0.56 | 0.90 |  | 0.84 | 0.40 |  | 0.83 | 0.97 | 0.93 | 0.85 |
| GGU(G) | 1.40 | 1.47 |  | 1.03 | 1.18 |  | 1.02 | 1.47 | 1.43 | **2.14** |
| GGC(G) | 0.22 | 0.19 |  | 0.37 | 0.54 |  | 0.24 | 0.13 | 0.21 | 0.31 |
| GGA(G) | **2.16** | **1.98** |  | **2.27** | **1.92** |  | **1.9** | **1.85** | **2.10** | 1.32 |
| GGG(G) | 0.22 | 0.35 |  | 0.33 | 0.37 |  | 0.83 | 0.55 | 0.26 | 0.23 |

Preferred codons for each P genotype were shown in bold.
