# Supplementary material for: Codon usage of host-specific P genotypes (VP4) in group A rotavirus
Source: BMC Genomics. 2022 Jul 16;23:518. doi: 10.1186/s12864-022-08730-2 (PMC9288207; doi:10.1186/s12864-022-08730-2)
Supplement: Supplementary file 3 — Additional file 3: Figure S1. Scree plot of percentage of explained variances for each principal component of the relative synonymous codon usage (RSCU) values of group A rotavirus VP4 coding sequences. This plot showed the proportion of variance in the RSCU values for each principal component (dimension), in descending order of magnitude. [file 12864_2022_8730_MOESM3_ESM.docx]

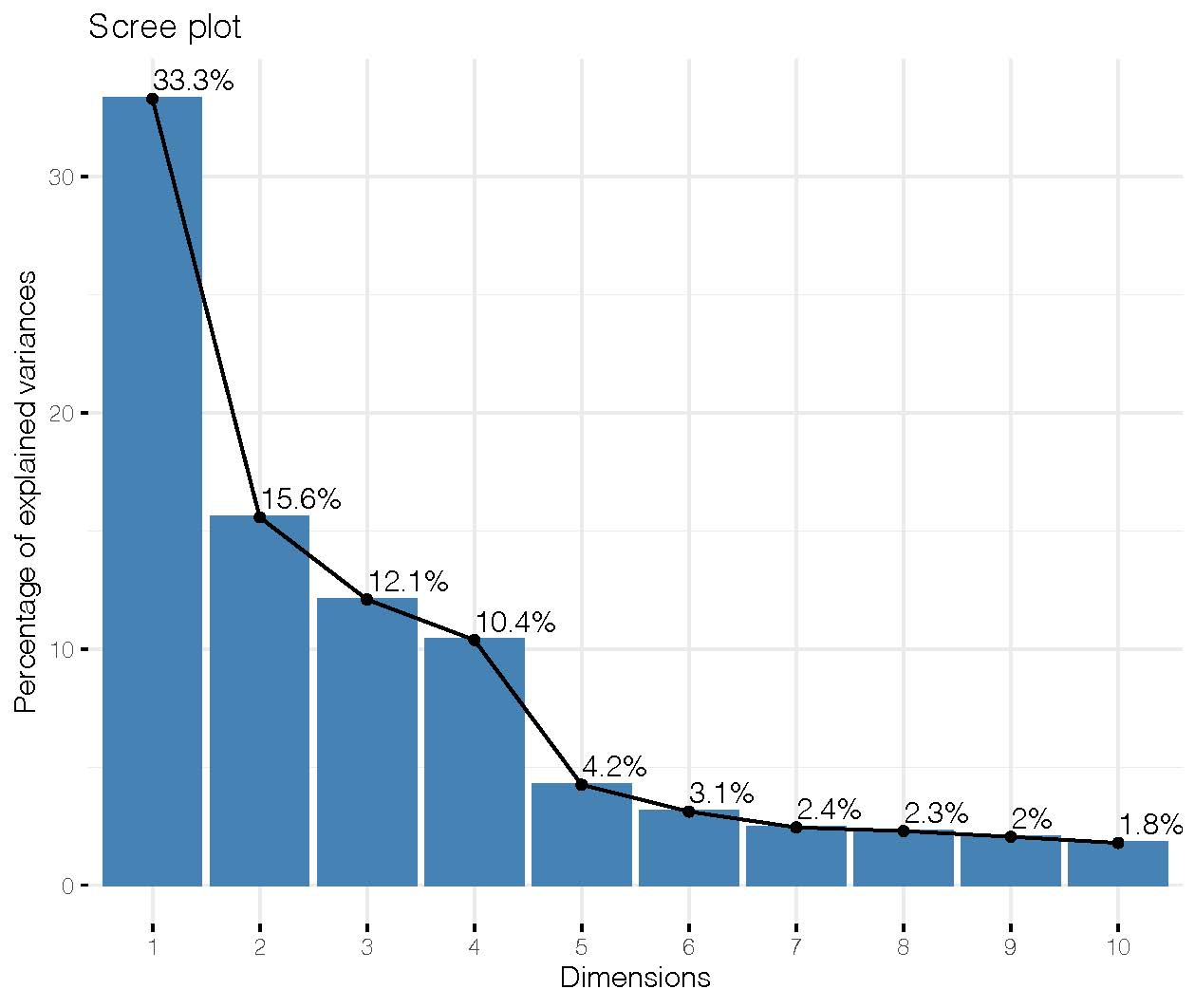


**Additional file 3: Figure S1.** Scree plot of percentage of explained variances for each principal component of the relative synonymous codon usage (RSCU) values of group A rotavirus VP4 coding sequences. This plot showed the proportion of variance in the RSCU values for each principal component (dimension), in descending order of magnitude.
