# Supplementary material for: Codon usage of host-specific P genotypes (VP4) in group A rotavirus
Source: BMC Genomics. 2022 Jul 16;23:518. doi: 10.1186/s12864-022-08730-2 (PMC9288207; doi:10.1186/s12864-022-08730-2)
Supplement: Supplementary file 4 — Additional file 4: Table S3. VP4 coding sequences for group A rotavirus P[4], P[8], P[13], P[23], P[1], P[6], P[7] and P[19]. [file 12864_2022_8730_MOESM4_ESM.docx]

**Table S3. VP4 coding sequences for group A rotavirus P[4], P[8], P[13], P[23], P[1], P[6], P[7] and P[19].**

| **Accession number** | **Strain name** | **P genotype** | **Host** | **Country** | **Collection Year** |
| --- | --- | --- | --- | --- | --- |
| KC178794 | PA150/2006 | P[4] | Homo sapiens | Italy | 2006 |
| KC178795 | PA83/2007 | P[4] | Homo sapiens | Italy | 2007 |
| KC178797 | PA17/2008 | P[4] | Homo sapiens | Italy | 2008 |
| KJ940070 | RVA/Human-wt/BRA/RS15851/2008/G2P[4] | P[4] | Homo sapiens | Brazil | 2008 |
| KJ940071 | RVA/Human-wt/BRA/ES16238/2009/G2P[4] | P[4] | Homo sapiens | Brazil | 2009 |
| KX536661 | RV09 | P[4] | Homo sapiens | India | 2009 |
| KX646613 | RV0904 | P[4] | Homo sapiens | India | 2009 |
| MT232574 | RVA/Human-wt/USA/2009727045/2009/G8P[4] | P[4] | Homo sapiens | USA | 2009 |
| KC178790 | PA130/2010 | P[4] | Homo sapiens | Italy | 2010 |
| KJ940072 | RVA/Human-wt/BRA/RJ17745/2010/G2P[4] | P[4] | Homo sapiens | Brazil | 2010 |
| KX536662 | RV10 | P[4] | Homo sapiens | India | 2010 |
| KX646614 | RV1011 | P[4] | Homo sapiens | India | 2010 |
| MT633452 | TO-073 | P[4] | Homo sapiens | Brazil | 2010 |
| MZ093649 | RVA/Human-wt/KEN/KLF0320/2010/G8P[4] | P[4] | Homo sapiens | Kenya: Kilifi | 2010 |
| KC178791 | PA133/2011 | P[4] | Homo sapiens | Italy | 2011 |
| KF716325 | RVA/Human-wt/USA/VU10-11-5/2011/G2P[4] | P[4] | Homo sapiens | USA: Vanderbilt | 2011 |
| KF716333 | RVA/Human-wt/USA/VU10-11-19/2011/G2P[4] | P[4] | Homo sapiens | USA: Vanderbilt | 2011 |
| KF716334 | RVA/Human-wt/USA/VU10-11-2/2011/G2P[4] | P[4] | Homo sapiens | USA: Vanderbilt | 2011 |
| KJ940075 | RVA/Human-wt/BRA/SC19868/2011/G2P[4] | P[4] | Homo sapiens | Brazil | 2011 |
| KX536663 | RV11 | P[4] | Homo sapiens | India | 2011 |
| KX646617 | RV1109 | P[4] | Homo sapiens | India | 2011 |
| KX646618 | RV1110 | P[4] | Homo sapiens | India | 2011 |
| KX646633 | RV1113 | P[4] | Homo sapiens | India | 2011 |
| KX362835 | RVA/Human-wt/VNM/12013_45/VP4 | P[4] | Homo sapiens | Viet Nam | 2012 |
| KX646621 | RV1206 | P[4] | Homo sapiens | India | 2012 |
| KX646623 | RV1208 | P[4] | Homo sapiens | India | 2012 |
| MT232618 | RVA/Human-wt/USA/2012748260/2012/G8P[4] | P[4] | Homo sapiens | USA | 2012 |
| MZ093680 | RVA/Human-wt/KEN/KLF0541/2012/G8P[4] | P[4] | Homo sapiens | Kenya: Kilifi | 2012 |
| MZ094016 | RVA/Human-wt/KEN/KLF0576/2012/G8P[4] | P[4] | Homo sapiens | Kenya: Kilifi | 2012 |
| MZ097182 | RVA/Human-wt/KEN/KLF1055/2012/G8P[4] | P[4] | Homo sapiens | Kenya: Kilifi | 2012 |
| KX362583 | RVA/Human-wt/VNM/16020_77/VP4 | P[4] | Homo sapiens | Viet Nam | 2013 |
| KX646624 | RV1306 | P[4] | Homo sapiens | India | 2013 |
| KX646626 | RV1308 | P[4] | Homo sapiens | India | 2013 |
| KX646640 | RV1315 | P[4] | Homo sapiens | India | 2013 |
| MG729831 | Hu/13-146/CHN/2013/GxP[4] | P[4] | Homo sapiens | China | 2013 |
| MT633447 | TO-002 | P[4] | Homo sapiens | Brazil | 2013 |
| MN478651 | RVA/Human-wt/USA/2014737624/2014/G2P[4] | P[4] | Homo sapiens | USA: Kansas City | 2014 |
| MN478657 | RVA/Human-wt/USA/2014741096/2014/G2P[4] | P[4] | Homo sapiens | USA: Houston | 2014 |
| MN478700 | RVA/Human-wt/USA/2014753259/2014/G2P[4] | P[4] | Homo sapiens | USA: Houston | 2014 |
| MN478707 | RVA/Human-wt/USA/3000347103/2014/G2P[4] | P[4] | Homo sapiens | USA: Cincinnati | 2014 |
| MT633448 | TO-022 | P[4] | Homo sapiens | Brazil | 2014 |
| MN478701 | RVA/Human-wt/USA/3000007730/2015/G2P[4] | P[4] | Homo sapiens | USA: Cincinnati | 2015 |
| MN478712 | RVA/Human-wt/USA/3000347195/2015/G2P[4] | P[4] | Homo sapiens | USA: Cincinnati | 2015 |
| MN478760 | RVA/Human-wt/USA/3000380081/2015/G2P[4] | P[4] | Homo sapiens | USA: Cincinnati | 2015 |
| MN478767 | RVA/Human-wt/USA/3000380681/2015/G2P[4] | P[4] | Homo sapiens | USA: Seattle | 2015 |
| MT633454 | TO-095 | P[4] | Homo sapiens | Brazil | 2015 |
| MN478796 | RVA/Human-wt/USA/3000558291/2016/G2P[4] | P[4] | Homo sapiens | USA: Seattle | 2016 |
| MT633450 | TO-048 | P[4] | Homo sapiens | Brazil | 2016 |
| MZ027435 | RVA/Human-wt/ZMB/UFS-NGS-MRC-DPRU13327/2016/G2P[4] | P[4] | Homo sapiens | Zambia | 2016 |
| MZ096555 | RVA/Human-wt/KEN/KLF0927/2019/G8P[4] | P[4] | Homo sapiens | Kenya: Kilifi | 2019 |
| KP902534 | RVA/Human-wt/MWI/OP354/1998/G4P[8] | P[8] | Homo sapiens | Malawi | 1988 |
| KP902547 | RVA/Human-wt/FIN/Fin-302/1988/G1P[8] | P[8] | Homo sapiens | Finland | 1988 |
| KP902533 | RVA/Human-wt/MWI/OP530/1999/G4P[8] | P[8] | Homo sapiens | Malawi | 1999 |
| KP902549 | RVA/Human-wt/ZAF/MRC-DPRU2144/2003/G9P[8] | P[8] | Homo sapiens | South Africa | 2003 |
| GQ869838 | DH389 | P[8] | Homo sapiens | Bangladesh | 2004 |
| EU979379 | MMC38 | P[8] | human | Bangladesh | 2005 |
| EU979382 | MMC71 | P[8] | human | Bangladesh | 2005 |
| FJ435210 | RVA/Human-wt/RUS/Nov06-1486/2006/G1P[8] | P[8] | Homo sapiens | Russia: Novosibirsk | 2006 |
| GQ869840 | MMC183 | P[8] | Homo sapiens | Bangladesh | 2006 |
| KF907291 | RVA/Human-wt/BRA/RJ12419/2006/G12P[8] | P[8] | Homo sapiens | Brazil | 2006 |
| KP902544 | RVA/Human-wt/ISR/R0659/2006/G9P[8] | P[8] | Homo sapiens | Israel | 2006 |
| KP902536 | RVA/Human-wt/JOR/J597/2007/G1P[8] | P[8] | Homo sapiens | Jordan | 2007 |
| KP902541 | RVA/Human-wt/ISR/R1909/2007/G9P[8] | P[8] | Homo sapiens | Israel | 2007 |
| GU320755 | Hu/RUS/Omsk08-381/2008/G9P[8] | P[8] | Homo sapiens | Russia: Omsk | 2008 |
| JF813104 | Z1108 | P[8] | Homo sapiens | China | 2008 |
| KP902560 | RVA/Human-wt/BEL/BE1014/2008/G4P[8] | P[8] | Homo sapiens | Belgium | 2008 |
| MT163246 | RVA/Human-wt/ZAF/UFS-NGS-MRC-DPRU1971/2008/G1P[8] | P[8] | Homo sapiens | South Africa | 2008 |
| GQ453422 | RVA/Human-wt/RUS/Nov09-D10/2009/G9P[8] | P[8] | Homo sapiens | Russia: Novosibirsk | 2009 |
| JF813100 | E1545 | P[8] | Homo sapiens | China | 2009 |
| JQ248944 | Hu/RUS/Nov09-D187/2009/G1P[8] | P[8] | Homo sapiens | Russia: Novosibirsk | 2009 |
| KP902539 | RVA/Human-wt/ISR/R5808/2009/G9P[8] | P[8] | Homo sapiens | Israel | 2009 |
| KP902553 | RVA/Human-wt/BEL/BE2003/2009/G1P[8] | P[8] | Homo sapiens | Belgium | 2009 |
| KP902554 | RVA/Human-wt/BEL/BE1418/2009/G9P[8] | P[8] | Homo sapiens | Belgium | 2009 |
| KX646551 | RV0901 | P[8] | Homo sapiens | India | 2009 |
| KX646555 | RV0910 | P[8] | Homo sapiens | India | 2009 |
| KX646556 | RV0911 | P[8] | Homo sapiens | India | 2009 |
| KX646605 | RV0908 | P[8] | Homo sapiens | India | 2009 |
| KX646606 | RV0909 | P[8] | Homo sapiens | India | 2009 |
| HQ537486 | Hu/RUS/Nov10-N53/2010/G4P[8] | P[8] | Homo sapiens | Russia: Novosibirsk | 2010 |
| JQ289056 | Hu/RUS/Nov10-N670/G4P[8] | P[8] | Homo sapiens | Russia: Novosibirsk | 2010 |
| KF648957 | Hu/RUS/Nov10-N736/2010/G1P[8] | P[8] | Homo sapiens | Russia: Novosibirsk | 2010 |
| KP902538 | RVA/Human-wt/ISR/R6293/2010/G9P[8] | P[8] | Homo sapiens | Israel | 2010 |
| KX646598 | RV1005 | P[8] | Homo sapiens | India | 2010 |
| KX646599 | RV1013 | P[8] | Homo sapiens | India | 2010 |
| KX646608 | RV1014 | P[8] | Homo sapiens | India | 2010 |
| KY497543 | RVA/Human-wt/PAK/42/2010/G1P8 | P[8] | Homo sapiens | Pakistan | 2010 |
| MT633400 | TO-074 | P[8] | Homo sapiens | Brazil | 2010 |
| MZ093660 | RVA/Human-wt/KEN/KLF0389/2010/G9P[8] | P[8] | Homo sapiens | Kenya: Kilifi | 2010 |
| JQ613163 | Hu/RUS/Nov11-N1936/2011/G2P[8] | P[8] | Homo sapiens | Russia: Novosibirsk | 2011 |
| JX435099 | Hu/RUS/Nov11-N2530/2011/G4P[8] | P[8] | Homo sapiens | Russia: Novosibirsk | 2011 |
| KF648958 | Hu/RUS/Nov11-N2476/2011/G4P[8] | P[8] | Homo sapiens | Russia: Novosibirsk | 2011 |
| KX646571 | RV1101 | P[8] | Homo sapiens | India | 2011 |
| KX646572 | RV1102 | P[8] | Homo sapiens | India | 2011 |
| KX646601 | RV1114 | P[8] | Homo sapiens | India | 2011 |
| MN632685 | RVA/Human-wt/RWA/UFS-NGS-MRC-DPRU1565/2011/G1P[8] | P[8] | Homo sapiens | Rwanda | 2011 |
| MN632762 | RVA/Human-wt/RWA/UFS-NGS-MRC-DPRU1609/2011/G1P[8] | P[8] | Homo sapiens | Rwanda | 2011 |
| MN632784 | RVA/Human-wt/RWA/UFS-NGS-MRC-DPRU1626/2011/G1P[8] | P[8] | Homo sapiens | Rwanda | 2011 |
| MT633416 | TO-092 | P[8] | Homo sapiens | Brazil | 2011 |
| KX362848 | RVA/Human-wt/VNM/12013_46/VP4_c1 | P[8] | Homo sapiens | Viet Nam | 2012 |
| KX646580 | RV1201 | P[8] | Homo sapiens | India | 2012 |
| KX646602 | RV1217 | P[8] | Homo sapiens | India | 2012 |
| MN632904 | RVA/Human-wt/RWA/UFS-NGS-MRC-DPRU442/2012/G1P[8] | P[8] | Homo sapiens | Rwanda | 2012 |
| MT633404 | TO-080 | P[8] | Homo sapiens | Brazil | 2012 |
| MZ093745 | RVA/Human-wt/KEN/KLF0548/2012/G1P[8] | P[8] | Homo sapiens | Kenya: Kilifi | 2012 |
| KX362594 | RVA/Human-wt/VNM/16020_35/VP4 | P[8] | Homo sapiens | Viet Nam | 2013 |
| KX363296 | RVA/Human-wt/VNM/12070_94/VP4 | P[8] | Homo sapiens | Viet Nam | 2013 |
| KX646587 | RV1301 | P[8] | Homo sapiens | India | 2013 |
| KX646593 | RV1326 | P[8] | Homo sapiens | India | 2013 |
| KX646603 | RV1313 | P[8] | Homo sapiens | India | 2013 |
| KX646604 | RV1314 | P[8] | Homo sapiens | India | 2013 |
| LC469338 | RVA/Human-wt/IDN/D37/2013/G1P[8] | P[8] | Homo sapiens | Indonesia: East Java | 2013 |
| MG816517 | human/SC11/CHN/2013/P[8] | P[8] | Homo sapiens | China: Chengdu | 2013 |
| MG816521 | human/SC2/CHN/2013/P[8] | P[8] | Homo sapiens | China: Chengdu | 2013 |
| MN632915 | RVA/Human-wt/RWA/UFS-NGS-MRC-DPRU568/2013/G1P[8] | P[8] | Homo sapiens | Rwanda | 2013 |
| MN632970 | RVA/Human-wt/RWA/UFS-NGS-MRC-DPRU714/2013/G1P[8] | P[8] | Homo sapiens | Rwanda | 2013 |
| MT633355 | TO-006 | P[8] | Homo sapiens | Brazil | 2013 |
| MT633356 | TO-007 | P[8] | Homo sapiens | Brazil | 2013 |
| MT633415 | TO-091 | P[8] | Homo sapiens | Brazil | 2013 |
| MZ094438 | RVA/Human-wt/KEN/KLF0632/2013/G1P[8] | P[8] | Homo sapiens | Kenya: Kilifi | 2013 |
| MZ094493 | RVA/Human-wt/KEN/KLF0637/2013/G1P[8] | P[8] | Homo sapiens | Kenya: Kilifi | 2013 |
| LC469340 | RVA/Human-wt/IDN/GRV67/2014/G1P[8] | P[8] | Homo sapiens | Indonesia: East Java | 2014 |
| MG816515 | human/SC9/CHN/2014/P[8] | P[8] | Homo sapiens | China: Chengdu | 2014 |
| MG816520 | human/SC1/CHN/2014/P[8] | P[8] | Homo sapiens | China: Chengdu | 2014 |
| MN478653 | RVA/Human-wt/USA/2014738127/2014/G1P[8] | P[8] | Homo sapiens | USA: Kansas City | 2014 |
| MN478669 | RVA/Human-wt/USA/2014741246/2014/G1P[8] | P[8] | Homo sapiens | USA: Houston | 2014 |
| MN478686 | RVA/Human-wt/USA/2014741572/2014/G1P[8] | P[8] | Homo sapiens | USA: Houston | 2014 |
| MN478696 | RVA/Human-wt/USA/2014751652/2014/G1P[8] | P[8] | Homo sapiens | USA: Houston | 2014 |
| MN478697 | RVA/Human-wt/USA/2014751657/2014/G1P[8] | P[8] | Homo sapiens | USA: Houston | 2014 |
| MN478704 | RVA/Human-wt/USA/3000014466/2014/G9P[8] | P[8] | Homo sapiens | USA: Seattle | 2014 |
| MT633420 | TO-114 | P[8] | Homo sapiens | Brazil | 2014 |
| MT633421 | TO-118 | P[8] | Homo sapiens | Brazil | 2014 |
| MT633430 | TO-181 | P[8] | Homo sapiens | Brazil | 2014 |
| MT633435 | TO-186 | P[8] | Homo sapiens | Brazil | 2014 |
| MZ027413 | RVA/Human-wt/ZMB/UFS-NGS-MRC-DPRU4749/2014/G2P[8] | P[8] | Homo sapiens | Zambia | 2014 |
| MZ097083 | RVA/Human-wt/KEN/KLF1046/2014/G1P[8] | P[8] | Homo sapiens | Kenya: Kilifi | 2014 |
| MZ097094 | RVA/Human-wt/KEN/KLF1047/2014/G1P[8] | P[8] | Homo sapiens | Kenya: Kilifi | 2014 |
| KX778573 | RVA/Human-wt/CHN/km15007/G9P[8] | P[8] | Homo sapiens | China: Kunming | 2015 |
| KX778576 | RVA/Human-wt/CHN/km15093/G9P[8] | P[8] | Homo sapiens | China: Kunming | 2015 |
| LC469325 | RVA/Human-wt/IDN/STM004/2015/G3P[8] | P[8] | Homo sapiens | Indonesia: East Java | 2015 |
| MN417972 | PAK274 | P[8] | Homo sapiens | Pakistan | 2015 |
| MN478705 | RVA/Human-wt/USA/3000053718/2015/G9P[8] | P[8] | Homo sapiens | USA: Vanderbilt | 2015 |
| MN478723 | RVA/Human-wt/USA/3000352507/2015/G12P[8] | P[8] | Homo sapiens | USA: Vanderbilt | 2015 |
| MN478732 | RVA/Human-wt/USA/3000354421/2015/G12P[8] | P[8] | Homo sapiens | USA: Houston | 2015 |
| MN478741 | RVA/Human-wt/USA/3000356580/2015/G12P[8] | P[8] | Homo sapiens | USA: Oakland | 2015 |
| MN478749 | RVA/Human-wt/USA/3000368549/2015/G9P[8] | P[8] | Homo sapiens | USA: Rochester | 2015 |
| MN478752 | RVA/Human-wt/USA/3000371148/2015/G1P[8] | P[8] | Homo sapiens | USA: Kansas City | 2015 |
| MN478761 | RVA/Human-wt/USA/3000380129/2015/G12P[8] | P[8] | Homo sapiens | USA: Cincinnati | 2015 |
| MN632674 | RVA/Human-wt/RWA/UFS-NGS-MRC-DPRU10007/2015/G1P[8] | P[8] | Homo sapiens | Rwanda | 2015 |
| MT633353 | TO-004 | P[8] | Homo sapiens | Brazil | 2015 |
| MZ095346 | RVA/Human-wt/KEN/KLF0755/2015/G9P[8] | P[8] | Homo sapiens | Kenya: Kilifi | 2015 |
| MZ095401 | RVA/Human-wt/KEN/KLF0764/2015/G1P[8] | P[8] | Homo sapiens | Kenya: Kilifi | 2015 |
| MZ095412 | RVA/Human-wt/KEN/KLF0765/2015/G1P[8] | P[8] | Homo sapiens | Kenya: Kilifi | 2015 |
| KX778583 | RVA/Human-wt/CHN/km15118/G9P[8] | P[8] | Homo sapiens | China: Kunming | 2016 |
| LC469330 | RVA/Human-wt/IDN/STM102/2016/G3P[8] | P[8] | Homo sapiens | Indonesia: East Java | 2016 |
| MN417973 | PAK439 | P[8] | Homo sapiens | Pakistan | 2016 |
| MN478724 | RVA/Human-wt/USA/3000353040/2016/G6P[8] | P[8] | Homo sapiens | USA: Vanderbilt | 2016 |
| MN478744 | RVA/Human-wt/USA/3000357125/2016/G9P[8] | P[8] | Homo sapiens | USA: Oakland | 2016 |
| MN478745 | RVA/Human-wt/USA/3000368400/2016/G9P[8] | P[8] | Homo sapiens | USA: Rochester | 2016 |
| MN478758 | RVA/Human-wt/USA/3000378272/2016/G1P[8] | P[8] | Homo sapiens | USA: Cincinnati | 2016 |
| MN478798 | RVA/Human-wt/USA/3000558351/2016/G12P[8] | P[8] | Homo sapiens | USA: Seattle | 2016 |
| MN632893 | RVA/Human-wt/RWA/UFS-NGS-MRC-DPRU16728/2016/G1P[8] | P[8] | Homo sapiens | Rwanda | 2016 |
| MZ027424 | RVA/Human-wt/ZMB/UFS-NGS-MRC-DPRU13232/2016/G1P[8] | P[8] | Homo sapiens | Zambia | 2016 |
| MZ027446 | RVA/Human-wt/ZMB/UFS-NGS-MRC-DPRU13541/2016/G1P[8] | P[8] | Homo sapiens | Zambia | 2016 |
| MZ097116 | RVA/Human-wt/KEN/KLF1049/2016/G1P[8] | P[8] | Homo sapiens | Kenya: Kilifi | 2016 |
| LC469336 | RVA/Human-wt/IDN/STM369/2017/G3P[8] | P[8] | Homo sapiens | Indonesia: East Java | 2017 |
| MZ097127 | RVA/Human-wt/KEN/KLF1050/2017/G1P[8] | P[8] | Homo sapiens | Kenya: Kilifi | 2017 |
| MZ096199 | RVA/Human-wt/KEN/KLF0877/2018/G1P[8] | P[8] | Homo sapiens | Kenya: Kilifi | 2018 |
| MZ097160 | RVA/Human-wt/KEN/KLF1053/2018/G1P[8] | P[8] | Homo sapiens | Kenya: Kilifi | 2018 |
| MW254173 | RVA/sewage/CHN/B16-R3/2019/P8 | P[8] | Homo sapiens | China | 2019 |
| MZ096610 | RVA/Human-wt/KEN/KLF0933/2019/G1P[8] | P[8] | Homo sapiens | Kenya: Kilifi | 2019 |
| KU048605 | Hu-wt/ITA/PA414/12/2012/G12P8 | P[8] | Homo sapiens | Italy | 2012 |
| KU048604 | Hu-wt/ITA/ME659/14/2014/G12P8 | P[8] | Homo sapiens | Italy | 2014 |
| KX363381 | RVA/Pig-wt/VNM/14225_45/VP4 | P[13] | Sus scrofa domesticus | Viet Nam | 2012 |
| MH238287 | RVA/Pig-wt/ESP/F456/2017/G5P[13] | P[13] | Sus scrofa | Spain | 2017 |
| MH238288 | RVA/Pig-wt/ESP/F471/2017/G3P[13] | P[13] | Sus scrofa | Spain | 2017 |
| KX363359 | RVA/Pig-wt/ESP/F35/2017/G9P[23] | P[23] | Sus scrofa domesticus | Viet Nam | 2012 |
| MH238267 | RVA/Pig-wt/ESP/F35/2017/G9P[23] | P[23] | Sus scrofa | Spain | 2017 |
| MH238269 | RVA/Pig-wt/ESP/F108/2017/G9P[23] | P[23] | Sus scrofa | Spain | 2017 |
| MH238270 | RVA/Pig-wt/ESP/F222/2017/G9P[23] | P[23] | Sus scrofa | Spain | 2017 |
| MH238273 | RVA/Pig-wt/ESP/F264/2017/G4P[23] | P[23] | Sus scrofa | Spain | 2017 |
| MH238274 | RVA/Pig-wt/ESP/F270/2017/G4P[23] | P[23] | Sus scrofa | Spain | 2017 |
| MH238275 | RVA/Pig-wt/ESP/F284/2017/G9P[23] | P[23] | Sus scrofa | Spain | 2017 |
| MH238276 | RVA/Pig-wt/ESP/F341/2017/G9P[23] | P[23] | Sus scrofa | Spain | 2017 |
| MH238279 | RVA/Pig-wt/ESP/F393/2017/G9P[23] | P[23] | Sus scrofa | Spain | 2017 |
| MH238280 | RVA/Pig-wt/ESP/F394/2017/G9P[23] | P[23] | Sus scrofa | Spain | 2017 |
| MH238283 | RVA/Pig-wt/ESP/F447/2017/G9P[23] | P[23] | Sus scrofa | Spain | 2017 |
| MH238284 | RVA/Pig-wt/ESP/F448/2017/G9P[23] | P[23] | Sus scrofa | Spain | 2017 |
| MH238285 | RVA/Pig-wt/ESP/F451/2017/G9P[23] | P[23] | Sus scrofa | Spain | 2017 |
| MH238286 | RVA/Pig-wt/ESP/F452/2017/G9P[23] | P[23] | Sus scrofa | Spain | 2017 |
| MH238289 | RVA/Pig-wt/ESP/F473/2017/G9P[23] | P[23] | Sus scrofa | Spain | 2017 |
| MH238290 | RVA/Pig-wt/ESP/F486/2017/G9P[23] | P[23] | Sus scrofa | Spain | 2017 |
| MH697616 | RVA/Pig-tc/CH/TM-a-P1/2018/G9P | P[23] | porcine | China | 2018 |
| AF361438 | HMG035 | P[1] | human | Nigeria | 2001 |
| MK638873 | RVA/Yak-tc/CHN/QH-1/2015/G6P[1] | P[1] | yak | China | 2015 |
| AF076925 | S12/85 | P[6] | human | us | 1998 |
| AF079356 | US1205 | P[6] | human | us | 1998 |
| MG570048 | porcine/Z84/CHN/2007 | P[6] | porcine | China: Hebei | 2007 |
| KX646642 | RV0915 | P[6] | Homo sapiens | India | 2009 |
| KF041444 | GX54 | P[6] | Homo sapiens | china | 2010 |
| KY497554 | RVA/Human-wt/PAK93/2010/G1P6 | P[6] | Homo sapiens | Pakistan | 2010 |
| KY748310 | RVA/Human-wt/THA/CMH-N016-10/2010/G4P[6] | P[6] | Homo sapiens | Thailand | 2010 |
| KX646645 | RV1115 | P[6] | Homo sapiens | India | 2011 |
| KY748311 | RVA/Human-wt/THA/CMH-N014-11/2011/G4P[6] | P[6] | Homo sapiens | Thailand | 2011 |
| KX363402 | RVA/Pig-wt/VNM/14226_39/VP4 | P[6] | Sus scrofa domesticus | Viet Nam | 2012 |
| KX646644 | RV1220 | P[6] | Homo sapiens | India | 2012 |
| KX646646 | RV1212 | P[6] | Homo sapiens | India | 2012 |
| KX646647 | RV1213 | P[6] | Homo sapiens | India | 2012 |
| KX646648 | RV1214 | P[6] | Homo sapiens | India | 2012 |
| KX646649 | RV1218 | P[6] | Homo sapiens | India | 2012 |
| KX646650 | RV1219 | P[6] | Homo sapiens | India | 2012 |
| KX646643 | RV1320 | P[6] | Homo sapiens | India | 2013 |
| MG729826 | Hu/13-10/CHN/2013/GxP[6] | P[6] | Homo sapiens | China | 2013 |
| MN478654 | RVA/Human-wt/USA/2014738139/2014/G3P[6] | P[6] | Homo sapiens | USA: Kansas City | 2014 |
| MT271026 | RVA/Human-wt/ZMB/UFS-NGS-MRC-DPRU4723/2014/G5P[6] | P[6] | Homo sapiens | Zambia | 2014 |
| MN478734 | RVA/Human-wt/USA/3000354444/2015/G3P[6] | P[6] | Homo sapiens | USA: Houston | 2015 |
| LC469332 | RVA/Human-wt/IDN/STM169/2016/G3P[6] | P[6] | Homo sapiens child | Indonesia: East Java | 2016 |
| LC469333 | RVA/Human-wt/IDN/STM182/2016/G3P[6] | P[6] | Homo sapiens child | Indonesia: East Java | 2016 |
| LC469334 | RVA/Human-wt/IDN/STM197/2016/G3P[6] | P[6] | Homo sapiens child | Indonesia: East Java | 2016 |
| MN478770 | RVA/Human-wt/USA/3000428941/2016/G3P[6] | P[6] | Homo sapiens | USA: Kansas City | 2016 |
| MN478785 | RVA/Human-wt/USA/3000526261/2016/G3P[6] | P[6] | Homo sapiens | USA: Vanderbilt | 2016 |
| MN478802 | RVA/Human-wt/USA/3000592598/2016/G3P[6] | P[6] | Homo sapiens | USA: Rochester | 2016 |
| LC469337 | RVA/Human-wt/IDN/ATM415/2017/G3P[6] | P[6] | Homo sapiens child | Indonesia: East Java | 2017 |
| MW254171 | RVA/sewage/CHN/B24-R2/2019/P6 | P[6] | Homo sapiens | China | 2019 |
| MW254172 | RVA/sewage/CHN/B15-R3/2019/P6 | P[6] | Homo sapiens | China | 2019 |
| KM820722 | RVA/Pig-tc/BEL/RV277/1977/G1P[7] | P[7] | Sus scrofa | Belgium | 1977 |
| KF500177 | KJ56-1 | P[7] | bovine | South Korea | 2004 |
| MH238268 | RVA/Pig-wt/ESP/F37/2017/G3P[7] | P[7] | Sus scrofa | Spain | 2017 |
| MH238271 | RVA/Pig-wt/ESP/F253/2017/G3P[7] | P[7] | Sus scrofa | Spain | 2017 |
| MH238272 | RVA/Pig-wt/ESP/F255/2017/G3P[7] | P[7] | Sus scrofa | Spain | 2017 |
| MH238278 | RVA/Pig-wt/ESP/F376/2017/G4P[7] | P[7] | Sus scrofa | Spain | 2017 |
| DQ887060 | NIV929893 | P[19] | Human | India | 2006 |
| MG407648 | RVA/Human-wt/BRA/rj24598/2015/G26P[19] | P[19] | Human | Brazil | 2015 |
| MH238281 | RVA/Pig-wt/ESP/F437/2017/G3P[19] | P[19] | Sus scrofa | Spain | 2017 |
